# Prevalence of Human Papillomavirus Variants and Genetic Diversity in the L1 Gene and Long Control Region of HPV16, HPV31, and HPV58 Found in North-East Brazil

**DOI:** 10.1155/2015/130828

**Published:** 2015-02-22

**Authors:** Ana Pavla Almeida Diniz Gurgel, Bárbara Simas Chagas, Carolina Medeiros do Amaral, Kamylla Conceição Gomes Nascimento, Lígia Rosa Sales Leal, Jacinto da Costa Silva Neto, Maria Tereza Cartaxo Muniz, Antonio Carlos de Freitas

**Affiliations:** ^1^Laboratory of Molecular Studies and Experimental Therapy (LEMTE), Department of Genetics, Federal University of Pernambuco, Avenida Professor Moraes Rego S/N, 50670-901 Recife, PE, Brazil; ^2^Department of Histology and Embryology, Federal University of Pernambuco, Avenida Professor Moraes Rego S/N, 50670-901 Recife, PE, Brazil; ^3^Molecular Biology Laboratory, Pediatric Oncohematology Center, University of Pernambuco, Avenida Agamenon Magalhaes S/N, 50100-130 Recife, PE, Brazil

## Abstract

This study showed the prevalence of human papillomavirus (HPV) variants as well as nucleotide changes within L1 gene and LCR of the HPV16, HPV31, and HPV58 found in cervical lesions of women from North-East Brazil.

## 1. Introduction

Cervical cancer is the second most significant cause of cancer in women worldwide, with more than 529,000 new cases diagnosed and 275,000 deaths in 2011 [1]. Among these, 85% of the total number of cervical cancer cases occur in developing countries [1]. In Brazil, cervical cancer is the third most common cancer among women [2].

It is well-established that persistent infections caused by human papillomavirus (HPV) is a key aetiological factor in the development of cervical lesions and cervical cancer [3]. To date, 184 HPV types have been identified (http://www.hpvcenter.se/html/refclones.html) and 62 belong to the* Alphapapillomavirus* genus. Among these, epidemiological data showed that HPV16 and HPV18 are responsible for 70% of cases of invasive cervical cancer worldwide [4]. Moreover, other* Alphapapillomavirus* genotypes, such as HPV31, HPV33, HPV35, HPV45, HPV52, and HPV58, are involved in 18% of cases of squamous cell carcinoma cancer worldwide [4].

Previous studies revealed that different variants of HPV16 coevolved with the three main human phylogenetic branches: African, Caucasian, and Asian [5, 6]. Hence, variants of HPV16 were grouped into five distinct categories spread across different geographical regions: Europe (E), Asia (As), Asian-America (AA), Africa 1 (AF-1), and Africa 2 (Af-2) [5, 6]. However, recent studies have redefined variant as a nucleotide sequence that differs by approximately 1% between two or more variants of the same HPV type [7–9]. In addition, sublineage also was redefined as a nucleotide sequence that differs from 0.5 to 0.9% within a full genome of the same HPV type [7–9]. Hence, according to this analysis the HPV16 has four variant lineages (A, B, C, and D) and nine sublineages; HPV31 showed three viral lineages (A, B, and C) and seven sublineages; and HPV58 has four variant lineages (A, B, C, and D) and seven sublineages [7].

Several studies have demonstrated that HPV16, HPV31, and HPV58 variants are associated with oncogenicity, persistence, and the progression of infection [6, 10–26]. Although there have been important studies about genomic diversity, there are few records of the variants of HPV16, HPV31, and HPV58 that are widespread in Brazil [21, 22, 27–30] and more specifically in North-East Brazil [31–33]. Previous studies have shown that HPV16, HPV31, HPV33, and HPV58 are the most common HPV types found in cervical samples from North-East Brazil [33–36]. For this reason, there is a need for studies concerning the genomic characterization of circulating HPV16, HPV31, and HPV58 variants due to their biological differences, which could explain at least part of the differences in infectivity and pathogenicity of some HPV variants. For instance, nucleotide changes in the L1 gene may affect its immune response to HPV16, HPV31, and HPV58. In addition, the polymorphic sites in long control region (LCR) may affect the transcriptional activity of E6 promoters [37].

Thus, the aim of this study was to detect nucleotide changes within L1 and LCR of HPV16, HPV31, and HPV58 in cervical samples obtained from North-East Brazil. In silico prediction of B-cell and T-cell epitopes in the L1 gene of HPV16, HPV31, and HPV58 was performed. Moreover, binding sites of transcriptional factors prediction were also performed in LCR of HPV16, HPV31, and HPV58. Finally, a phylogenetic analysis was conducted to determine which variants of HPV16, HPV31, and HPV58 are found in North-East Brazil.

## 2. Materials and Methods

### 2.1. Study Population and Ethics Statement

A total of 206 samples were randomly collected from women during their medical consultation at two locations: the Gynecological Unit of the Integrated Medicine Center, in Sergipe State, and Salgadinho Medical Care Center, in Maceió, Alagoas State, North-East Brazil, between November 2010 and July 2011. The study included women with low-grade intraepithelial lesions (LSIL) and high-grade intraepithelial lesions (HSIL). This study was approved by the Ethics Committee of the University of Alagoas (UFAL 004650/2010-55) and the Ethics Committee of the Federal University of Sergipe (CEP/CCS/UFPE N° 491/11).

### 2.2. Nucleic Acid Isolation and Detection of HPV Types 16, 31, and 58

The cervical cells were collected by using cytobrush and placed in polyethylene tubes containing phosphate-buffered saline and transferred to the Molecular Studies and Experimental Therapy Laboratory (LEMTE) and stored at −80°C until analysis. Nucleic acids were extracted by means of the DNeasy Blood and Tissue Kit 135 (Qiagen), in accordance with the manufacturer's instructions. A polymerase chain reaction (PCR) was performed with the* MDM2* gene to avoid the false negative and to assess the quality of the extracted DNA. Positive HPV16, HPV31, and HPV58 DNA were detected by using PCR with degenerate primers MY09/11 followed by direct sequencing. The positive HPV DNA was purified with the Invisorb Fragment Cleanup (Invitek) Kit and sequenced (in duplicate) by using BigDyeTM Terminator Cycle Sequencing Read Reaction Kit (Applied Biosystems) and ABI PRISM (Applied Biosystems) to obtain both the forward and reverse sequences.

### 2.3. Analysis of L1 Gene and LCR of HPV16, HPV31, and HPV58 by PCR and Sequencing

HPV16 (L1 *n* = 14, LCR *n* = 22), HPV31 (L1 *n* = 4, LCR *n* = 8), and HPV58 (L1 *n* = 8, LCR *n* = 5) found in cervical samples were further characterized by amplification of partial sequence of L1 and LCR by means of the specific primer pairs described in Table 1. The reactions were performed with a final volume of 25 *µ*L containing 50 ng of DNA, 20 pmol of each primer, and 1X PCR Master Mix (Promega). The PCR cycling conditions were as follows: initial denaturation at 95°C for 5 minutes, 35 cycles of denaturation at 95°C for 30 seconds, annealing at 56°C for 1 minute, elongation at 72°C for 2 minutes, and a final extension at 72°C for 10 minutes. The PCR products were run on the agarose gel (1%). Following this, the amplicons were purified with the Invisorb Fragment Cleanup (Invitek) Kit and nucleotide sequences were obtained by means of fluorescent BigDyeTM Terminator Cycle Sequencing using v 3.1 Ready Reaction ABI PRISM (Applied Biosystems) to obtain both the forward and reverse sequences. PCR and sequencing were performed in duplicate.

### 2.4. Data Analysis

The obtained sequences were assembled by means of the Staden package [38]. They were then evaluated to determine the nucleotide divergence relative to the nucleotide sequences of HPV16 (K02718), HPV31 (J04353), and HPV58 (D90400). Sequence comparisons were carried out using the Basic Local Alignment Search Tool (BLAST) and multiple alignments were performed by using the CLUSTALW (Mega 5.2, Beta version) program [39].

The Neighbor-Joining algorithm and the Kimura 2-Parameter model trees, with 1000 bootstrapped replicates, were built by using the MEGA package, version 5.2 [39]. Phylogenetic analyses were performed with LCR sequences of HPV16, HPV31, and HPV58. The partial sequence of L1 and LCR genes of the HPV16, HPV31, and HPV58 was deposited in the NCBI GenBank database, under the following accession numbers: HPV16 L1 gene: KJ467225-467238; HPV16 LCR: KJ452220-452242; HPV31 L1 gene: KJ452216-452219; HPV31 LCR: KJ435060-435067; HPV58 L1 gene: KJ467239-477246; HPV58 LCR: KJ567247-467252. The references for the viral sequences used to construct the phylogenetic branches were collected from the GenBank sequence database and are listed in Table 2.

### 2.5. B-Cell and T-Cell Epitope Prediction

The putative impact of the HPV variants was estimated in silico by predicting the B-cell and T-cell epitopes. In this study, it was assumed that changes in the amino acid sequences of L1 proteins within the B-cell epitope regions could affect the binding affinities of the neutralizing antibodies and in the case of the T-cell did not initiate an epitope-specific immune response. Thus, the B-cell epitope of prototype sequences was predicted by means of the BcePred server, which is available from URL: http://www.imtech.res.in/raghava/bcepred/. The prediction was carried out with the aid of physicochemical parameters, such as hydrophilicity, flexibility/mobility, accessibility, polarity, exposed surface, turns and antigenic propensity [40].

The T-cell epitope predictions were performed by using ProPred and ProPred I servers. The ProPred I server (available from URL: http://www.imtech.res.in/raghava/propred1/) was used to predict MHC Class-I binding regions [41], while the ProPred server, (available from URL: http://www.imtech.res.in/raghava/propred/) was used to predict MHC Class-II binding peptide [42].

### 2.6. Transcription Factor in the Binding Sites Prediction

The PROMO server (available from URL: http://alggen.lsi.upc.es/cgi-bin/promo_v3/promo/promoinit.cgi?dirDB=TF_8.3) was used to search within the LCR of the HPV16, HPV31, and HPV58 for potential binding sites for cellular and viral transcriptional factors. The transcriptional factors analyzed were the following: AP-1, E2, NF-1, Oct-1, YY1, C/EBP, and Sp1. Transcriptional factors were predicted within a dissimilarity margin of less than or equal to 15% [43, 44].

## 3. Results

### 3.1. Characteristics of the Population

**Figure 1:**
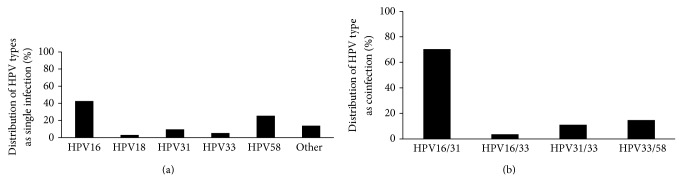
HPV type prevalence in North-East Brazil. (a) Distribution of HPV type as a single infection found in cervical lesions of women from North-East Brazil. (b) Distribution of HPV type as a coinfection found in cervical lesions of women from North-East Brazil.

A total of 206 cervical smear tests were carried out to detect HPV DNA and the results showed that 121 (59%) were positive for HPV. Among these, 94/121 (77.7%) were infected with one HPV type and 27/121 (22.3%) were infected with more than one HPV type (Figure 1). As a single infection, 40/94 (42.6%) samples were positive for HPV16, 3/94 (3.1%) samples were HPV18, 9/94 (9.6%) samples were positive for HPV31, 5/94 (5.3%) samples were HPV33, and 24/94 (25.5%) samples were positive for HPV58. A total of 13/94 (13.9%) of the samples were infected by other HPV types (Figure 1). With regard to coinfection, 19/27 (70.4%) samples were positive for HPV16/31, 1/27 (3.7%) sample was positive for HPV16/33, 3/27 (11.1%) samples were HPV31/33, and 4/27 samples (14.8%) were infected with HPV31/58 genotypes (Figure 1). Positive samples of HPV16, HPV31, and HPV58 were separated from the total, for molecular characterization.

### 3.2. HPV16

1300 pb nucleotide sequences of HPV16 L1 gene (*n* = 14) were compared with the reference sequence (K02718.1). DNA sequence analysis revealed twenty-three single nucleotide changes in the L1 gene, in which 10/23 (43.5%) showed nonsynonymous variations. The C6240G variation, which leads to H228D in the EF loop of the L1 protein, was observed in 100% of the samples and is embedded in B-cell and T-cell epitopes. Similarly, the insertion of ATC as well as the deletion of GAT, which leads to 447 threonine/448 serina and 445 aspartate amino acid changes respectively, was observed in all of the samples. These amino acid changes are located in the H4 and B-J regions and are embedded in T-cell epitopes. The nonsynonymous variations C5862T (H102Y), C6163A (T202N), A6176C (N207T), A6436G (T292A), and A6697C (T379P) are located in the EF, FG, and HI external loops and are embedded in B-cell and/or T-cell epitopes. Compared with the prototype of the HPV sequences, there was no evidence of premature stop codons in the HPV16 L1 gene variants. The detected variations are summarized in Table 3.

With regard to the HPV16 LCR sequences, thirty nucleotide changes were observed, in which 24/30 (80%) are embedded in the binding sites of transcriptional factors. The most common variations, insC7434, C7436G and delA7869, were found in 100% of the samples and are embedded in the YY1, NF-1, and E2 binding sites of the transcriptional factors, respectively. Moreover, the G7521A variation was found in 10/23 (43.5%) of the total number of samples, followed by A7489C (39.1%), G7493A (39.1%), C7693A (39.1%), C7768T (39.1%), and C7790T (39.1%). Among these variations, C7693A and C7790T are embedded in the E2 and YY1 binding sites, respectively. Some of these nucleotide changes are “diagnostic SNPs” that are conducted to detect the lineages and sublineages of HPV16 [15]. The detected variations are summarized in Table 4.

**Figure 2:**
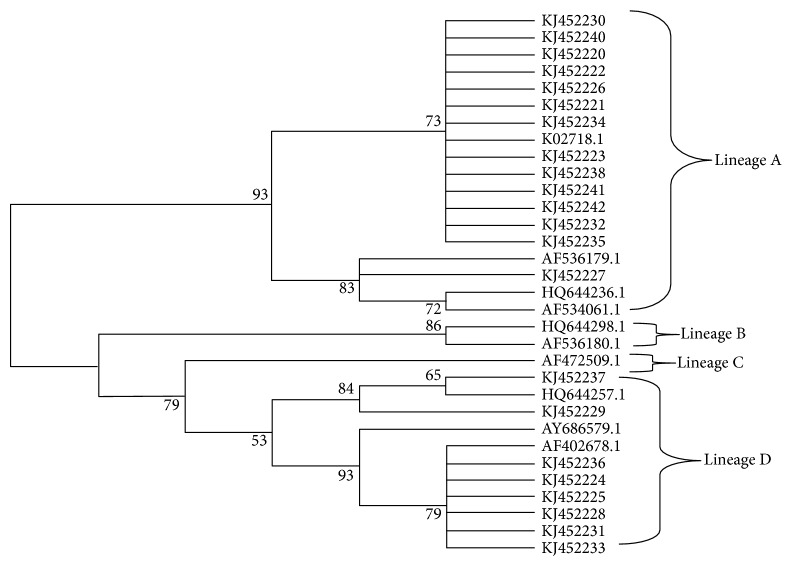
Neighbor joining phylogenetic tree of HPV16 variants based on 759 pb of LCR. Four clusters were identified as lineages A, B, C, and D. Reference sequences are listed in Table 2. Viral lineages analyzed in this study (KJ452220-452242) are clustered into A and D branches. Only bootstrap values above 50% are represented in the branches.

The phylogenetic analyses showed that 63.63% (14/22) samples belong to the A variant and 36.36% (8/22) samples belong to the D variant (Figure 2). No variants in the B or C variant were found in this study (Figure 2).

### 3.3. HPV31

The HPV31 L1 gene was analyzed through an alignment of 809 pb nucleotide sequences. The DNA sequence study revealed nine nucleotide changes in the L1 gene, two of which (2/9) were nonsynonymous variations and 7/9 were synonymous variations. When compared with the L1 protein of HPV16, the variations of A6025G (T267A) are located in the FG loop and embedded in the T-cell epitopes. Moreover, the C6379A nucleotide (T274N) is embedded in the FG loop as well as within the B-cell and T-cell epitopes. Compared with the prototype HPV sequences (J04353.1), there was no evidence of premature stop codons in the HPV31 L1 gene of variants. The detected variations are summarized in Table 5.

With regard to the HPV31 LCR nucleotide sequence, fragments of 883 pb were analyzed. Among these, thirty nucleotide changes were observed, 14/30 (47%) of which are embedded in the binding sites of the transcriptional factors. The most common variations were a deletion of TGTTCCTGCT at positions 7341–7450 (8/8, 100%) and located within the transcriptional binding sites of NF-1. C7480T and T7871G were found in 20% of the samples and are located within the binding sites of the E2 transcriptional factor. The detected variations are summarized in Table 6.

**Figure 3:**
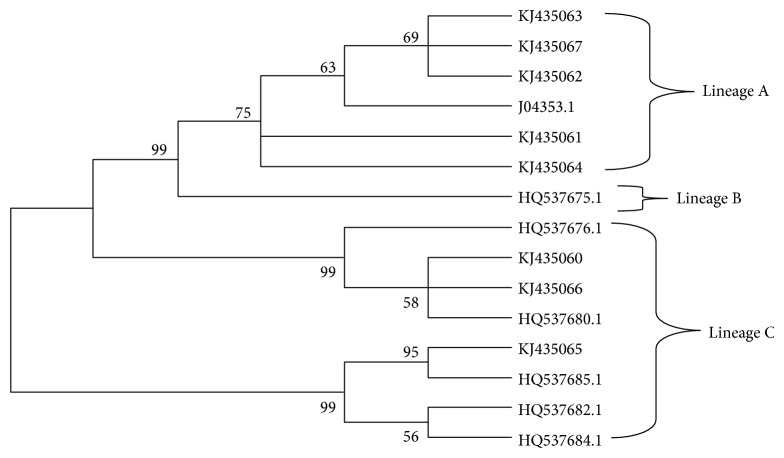
Neighbor joining phylogenetic tree of HPV31 variants based on 783 pb of LCR. Reference sequences are listed in Table 2. Three clusters were identified as lineages A, B, and C. Viral lineages analyzed in this study (KJ435060-KJ435067) are clustered into A and C branches. Only bootstrap values above 50% are represented in the branches.

The phylogenetic analysis showed 62.5% of variants are clustered into A branches (*n* = 5) and 37.5% are clustered into C branches (*n* = 3) (Figure 3). However, there were no observed variants clustered into B branches.

### 3.4. HPV58

The HPV58 L1 gene was analyzed through an alignment of the 1264 pb nucleotide sequence. Altogether, thirty-five single nucleotide polymorphisms were found, seven of which (7/35) were nonsynonymous variations. The most common are nucleotide changes were A6540G (I335M), C6828A (N422D), A6014C (L150F), G5994A (V144I), A6799G (I412V), G6823A (D420N), and C6689A (T375N); these are either located in the external loop (DE/HI loop) or alpha helix (H2 and H3) regions. Moreover, these polymorphisms are embedded in B-cell and/or T-cell epitopes. Compared with the prototype HPV sequence (D90400.1), insertion and deletion events were not identified and there was no evidence of premature stop codons or nucleotide deletions in the L1 HPV58 sequences analyzed. The detected variations are described in Table 7.

With regard to the HPV58 LCR sequences, thirty-five nucleotide changes were observed, in which 12/35 are embedded within the binding sites of transcriptional factors. The most common variations C7745A and A7794G were found in 50% of the samples and are embedded within the NF-1 and E2 binding sites of transcriptional factors, respectively. The detected variations are summarized in Table 8.

**Figure 4:**
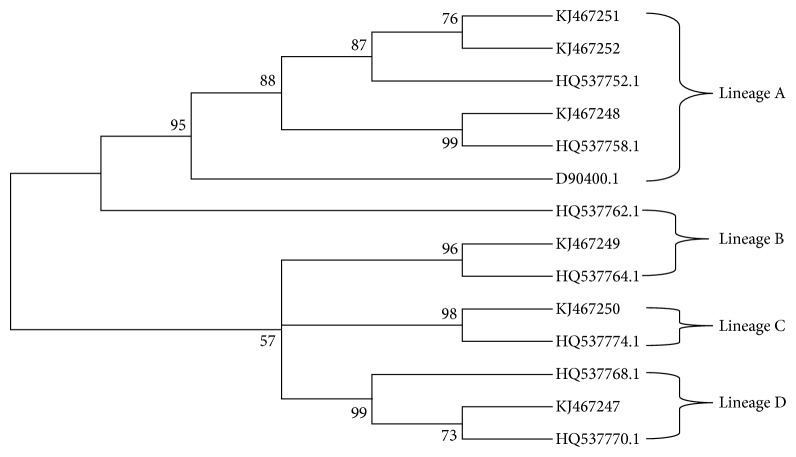
Neighbor joining phylogenetic tree of HPV58 variants based on 696 pb of LCR. Four clusters were identified as lineages A, B, C, and D. Reference sequences are listed in Table 2. Viral lineages analyzed in this study (KJ467247-KJ467252) are clustered into A, B, C, and D branches. Only bootstrap values above 50% are represented in the branches.

In addition, the phylogenetic analyses showed 50% of isolates belong to the A variant, followed by B (16.6%), C (16.6%), and D (16.6%) variants (Figure 4).

## 4. Discussion

Several studies have demonstrated that variants of HPV16, HPV31, and HPV58 may affect the oncogenicity, persistence, and progression of viral infection [6, 10–22, 24–26]. In this study, we evaluated the genetic diversity within L1 and LCR of HPV16, HPV31, and HPV58 in cervical samples from North-East Brazil. With regard to the HPV16, 23 nucleotide changes in L1 gene and 30 nucleotide changes in LCR were found. In addition, 9 nucleotide changes were found in L1 gene of HPV31 and 30 nucleotide changes also were found in LCR of HPV31. Moreover, 35 nucleotide changes in the L1 gene and LCR of HPV58 were found. Some of these nucleotide changes are putatively found in T-cell or B-cell epitope and in binding sites of transcriptional factor. Furthermore, two nucleotide changes in LCR of HPV31 and one deletion of seven base pair in LCR of HPV58 were described for the first time in this study. As far as we are aware, this is the first study of the genetic diversity of HPV16, HPV31, and HPV58 L1 and LCR in cervical samples from North-East Brazil.

Nucleotide changes within the HPV16 L1 gene can play an important role in the structure of the capsid protein, immune recognition, and viral neutralization [45]. Hence, viral polymorphisms in the L1 gene can affect the self-assembly of L1 protein in virus-like particles (VLPs) [46]. As a result, Kirnbauer et al. demonstrated that nucleotides change C6240G, and this leads to a change in the amino acid at position H202D, which is self- assembled within the VLPs with more efficiency in a heterologous system than with a prototype sequence [47]. In addition, it was found that variations in the 83–97 residues of the L1 gene have an impact on the yield of the L1 protein [48]. The nonsynonymous variations found in the L1 gene of HPV16, HPV31, and HPV58 of this study were reported in previous studies [49–58]. Some of these polymorphisms are located within hypervariable immuno-dominant regions (BC, DE, EF, FG, and HI loops) of L1 protein, which can be recognized as conformational epitopes of HPV [59, 60]. For instance, the A6436G polymorphisms (T292A) found in HPV16 and A6025G (T267A) and C6379A (T274N) found in HPV58 L1 genes are located in the FG loop of the L1 protein. In addition, the A6697C polymorphism (T379P) of HPV16 and C6689A (T375N) of HPV58 are located within the HI loop. Both the FG and HI loop constitute the immunodominant epitope region [61]. Furthermore, the polymorphisms found in helix 4 (including threonine and serine at 448 and 465 of L1 protein of HPV16) are implicated in the VLP formation [60].

Nucleotide variation within LCR may influence the binding affinity of the cellular and viral transcriptional factor. For instance, nucleotide changes may result in a loss or insertion of transcriptional factors that regulate the transcription of the of HR-HPV genes [62]. Hence, nucleotide changes in LCR of specific variants of HPV16, HPV31, and HPV58 may be involved in the alteration in the E6 and/or E7 oncogenes expression which could explain the potential carcinogenesis of some variants [62]. Some of the variations reported in this work are embedded in the putative binding sites for E2, C/EBPbeta, YY1, AP-1, NF-1, and Oct-1 transcriptional factors. These viral and cellular transcriptional factors are involved with early viral genes and differentiation of the epithelium, respectively. Hence, the nucleotide changes found in LCR of HPV16, HPV31, and HPV58 could be an impact directly or indirectly in the expression of E6 and E7 oncogenes.

In addition, we performed a phylogenetic analysis of HPV16 by using fragments of LCR. The results showed 63.63% of isolates belong to the A variant and 33.36% belong to the D variant for HPV16. These results are similar to the previous study performed in Central-West Brazil [63], which showed high prevalence of A and D variants and low frequency of B and C variants. In contrast, a recent study performed in South-Eastern Brazil showed A and C variants as the most prevalent, followed by D and B variants [64]. A previous study in 27 countries and using 953 cervical samples showed the A variant as the most prevalent, followed by C, B, and D variants [8]. These differences in the prevalence of HPV16 variants in different regions of Brazil and worldwide may be explained by geographic origin and ethnicity of the infected patients.

The LCR contains more phylogenetic information than other regions of the HPV16 genome and can distinguish both the lineages and sublineages [8]. Due to the lineage fixation and a putative nonrecombination process, studies have proposed diagnostic polymorphisms to classify both HPV16 lineages and sublineages [8, 9]. Cornet et al. proposed that variant lineages could be detected by using 32 SNP combinations in the LCR of HPV16 [8]. In the light of this, some of these diagnostic SNPs were found in the present study. For instance, the T7747G found in seven isolates of this study are diagnostic SNPs for the AA1 sublineage. Furthermore, G7891G found in seven isolates are diagnostic SNPs for the AA2 sublineage. Both AA1 and AA2 sublineages belong to lineage D [8].

With regard to the HPV31, the phylogenetic trees showed the presence of A and C variants in the North-East Brazil. These results are similar to the results obtained by Chagas et al., which reported high prevalence of A and C variants and very low prevalence of B variant in North-East Brazil [31, 32]. In addition, a recent study performed in Northern China also showed high prevalence of A and C variants [65]. In this study, we did not find any variants that belong to variant B, which was probably due to the small number of isolates analysed or the low frequency of this isolate in the North-East Brazil.

With regard to HPV58, variants that belong to the A, B, C, and D variants were found in North-East Brazil. In this study, A variant was the most prevalent (50%), followed by B (16.6%), C (16.6%), and D (16.6%) variants. In contrast, variant distribution worldwide and in the American continent showed the A variant as the most prevalent, followed by C, D, and E variants [66]. Additional studies should be performed to clarify whether these differences in prevalence of HPV58 variants are due to small number of isolates analysed or differences in prevalence of HPV58 variants in North-East Brazil.

In summary, this study reported the prevalence of HPV16, HPV31, and HPV58 variants and sequence variations in the L1 gene and LCR of HPV16, HPV31, and HPV58 isolates from North-East Brazil. Some of the polymorphisms found in the L1 gene are embedded within B-cell or T-cell epitopes. Moreover, some of the variations found in LCR are located within binding sites of transcriptional factors. Further studies should be carried out to throw light on both the pathological differences and the prevalence of these variants in different geographical regions.

## Figures and Tables

**Table 1:** Primers for HPV16, HPV31, and HPV58 L1 gene and LCR analysis.

HPV type	Region amplified	Sequence primer 5′-3′	Amplicon size
HPV16	L1	5′-ACGGTACCCAGGTGACTTTTATTTACATCC-3′	1500 pb
		5′-TAAGTCGACCAGCTTACGTTTTTTGC-3′	
	LCR	5′-TTCTGCAGACCTAGATCAGTTTC-3′	1057 pb
		5′-GTGCATAACTGTGGTAACTTTCTGG-3′	

HPV31	L1	5′-GATTCGAAAAAATGTCTCTGTGGC-3′	1550 pb
		5′-TAAGTCGACCTTTTTAGTTTTTTTACG-3′	
	LCR	5′-TACCTCCAAAGGAAAAGGAAGACCC-3′	1152 pb
		5′-TTGGCACAAATCATGCAATGTTCG-3′	

HPV58	L1	5′-AACCTGGTCCAGACATTGCATC-3′	1574 pb
		5′-CCACCAAACGCAAAAAGGTTA-3′	
	LCR	5′-CATGTTCTATGTCCTTGTCAG-3′	1000 pb
		5′-TTGCCAGGTGTGGACTAA-3′	

**Table 2:** Reference sequences used in phylogenetic analysis.

Reference sequences (GeneBank accession number)	HPV type/lineage	References
K02718 AF536179 HQ644236 AF534061 AF536180 HQ644298 AF472509 HQ644257 AY686579 AF402678	HPV16/A HPV16/A HPV16/A HPV16/A HPV16/B HPV16/B HPV16/C HPV16/D HPV16/D HPV16/D	(Burk et al., 2013) [7]

J04353 HQ537675 HQ537676 HQ537680 HQ537682 HQ537684 HQ537685	HPV31/A HPV31/A HPV31/B HPV31/B HPV31/C HPV31/C HPV31/C	(Burk et al., 2013) [7]

D90400 HQ537752 HQ537758 HQ537762 HQ537764 HQ537774 HQ537768 HQ537770	HPV58/A HPV58/A HPV58/A HPV58/B HPV58/B HPV58/C HPV58/D HPV58/D	(Burk et al., 2013) [7]

**Table 3:** HPV16 L1 sequence variations at the genome and protein level and the putative biological function affected.

Genome position	Variation	AA changed	Protein position	Number of isolates	Biological function
5862	C/T	H102Y	*β*-C	5/14	MHC Class-I/MHC Class-II
5909	T/C	—		5/14	
5981	G/A	—		1/14	
6023	A/T	—		1/14	
6163	C/A	T202N	EF-loop	5/14	B-cell/MHC Class-I
6176	A/C	N207T	EF-loop	2/14	B-cell/MHC Class-I/MHC Class-II
6240	C/G	H228D	EF-loop	14/14	B-cell/MHC Class-I
6245	T/C	—		5/14	
6300	A/C	—		1/14	
6315	A/G	—		4/14	
6436	A/G	T292A	FG-loop	5/14	MHC Class-I/MHC Class-II
6484	T/C	—		1/14	
6561	C/T	S308P		5/14	
6570	T/G	—		1/14	
6697	A/C	T379P	HI-loop	5/14	B-cell/MHC Class-I
6723	G/A	—		5/14	
6805	A/T	T415S	h2	1/14	MHC Class-I/MHC Class-II/VLP formation
6856	C/T	—		5/14	
6867	C/T	—		5/14	
6903	Ins ATC	Ins T and S 448	h4	14/14	MHC Class-I/
6955	Del GAT	Del D 465	h4-*β*-J		MHC Class-II/VLP formation
6972	C/T	—		5/14	
6996	G/A	—		5/14	

(—) Synonymous variations with regard to the prototype reference K02718.1. Del: deletion,  Ins: insertion,  H: histidine,  Y: tyrosine,  T: threonine,  N: asparagine,  D: aspartic acid,  A: alanine,  S: serine,  P: proline,  and Q: glutamine.

**Table 4:** HPV16 LCR sequence variations with regard to the reference sequence K02718.1. The table also shows the putative transcriptional factor binding sites in the nucleotide altered.

Genome position	Nucleotide variation	Number of isolates	Transcriptional factor
7175	A/C	1/23	C/EBPbeta
7194	G/T	10/23	
7229	A/C	1/23	C/EBPbeta
7234	A/C	10/23	
7258	G/A	1/23	YY1
7281	G/T	1/23	
7316	A/C	1/23	YY1, AP-1
7340	A/T	7/23	C/EBPbeta
7360	A/G	1/23	
7361	G/C	1/23	
7395	C/T	7/23	C/EBPbeta
7434	Ins C	23/23	NF-1, YY1
7436	C/G	23/23	YY1
7441	T/G	1/23	YY1
7485	A/C	9/23	NF1
7490	G/A	9/23	
7521	G/A	10/23	YY1
7670	C/T	9/23	
7690	C/A	9/23	E2
7730	A/C	8/23	Oct-1
7730	A/T	1/23	Oct-1
7747	T/G	7/23	C/EBPbeta
7768	C/T	9/23	NF-1
7790	C/T	1/23	YY1
7790	C/T	9/23	YY1
7838	G/T	2/23	C/EBPbeta
7869	Del A	23/23	E2
7874	G/A	1/23	NF-1
7881	C/A	1/23	C/EBPbeta
7891	C/G	7/23	C/EBPbeta

**Table 5:** HPV31 L1 sequence variations at the genome and protein level and the putative biological function affected.

Genome Position	Variation	AA changed	Protein position (relative to HPV16 L1 gene)	Number of isolates	Biological function
5926	A/G	—		1/4	
5927	T/C	—		1/4	
6025	A/G	T267A	FG loop	1/4	MHC Class-I/HC Class-II
6091	C/T	—		1/4	
6335	G/A	—		1/4	
6357	A/G	—		1/4	
6374	C/T	—		1/4	
6379	C/A	T274N	FG loop	1/4	B-cell/MHC Class-I
6593	T/G	—		1/4	

(—) Synonymous variations with regard to the reference sequence J04353.1. T: threonine; N: asparagine; A: alanine.

**Table 6:** HPV31 LCR sequence variation with regard to the reference sequence J04353.1. The table also shows the transcriptional factor binding sites in the nucleotide altered.

Genome position	Nucleotide variation	Number of isolates	Transcriptional factor
7157	A/G	1/8	YY1
7190	T/A^*^	1/8	C/EBPbeta
7305	Del CTATTTTATA	2/8	
7311	T/C	1/8	
7334	T/C	3/8	
7338	A/G	7/8	
7341	Del TGTTCCTGCT	8/8	NF-1
7360	A/G	3/8	AP-1, NF-1
7375	T/C	1/8	
7378	G/C	3/8	
7389	A/C^*^	1/8	
7390	G/A	5/8	
7400	C/A	3/8	YY1, NF-1
7455	G/A	3/8	YY1
7456	A/C	2/8	YY1
7463	G/A	3/8	
7476	A/C	1/8	
7480	C/T	2/8	E2
7512	C/T	2/8	C/EBPbeta
7521	C/A	1/8	C/EBPbeta
7531	G/A	3/8	
7539	T/C	1/8	E2
7581	T/C	3/8	NF-1
7648	C/A	1/8	AP-1
7665	T/G	1/8	
7713	A/G	1/8	
7716	C/T	2/8	
7755	A/C	2/8	
7760	C/A	2/8	
7871	T/G	2/8	E2

Del: deletion. ^*^Novel HPV31 mutation.

**Table 7:** HPV58 L1 sequence variations at the genome and protein level and the putative biological function affected.

Genome position	Variation	AA changed	Number of isolates	Protein position relative to HPV16 L1 gene	Biological function
5593	C/T	—	2/8		
5747	T/C	—	1/8		
5789	T/C	—	1/8		
5801	A/G	—	1/8		
5819	A/G	—	1/8		
5861	C/G	—	1/8		
5939	G/A	—	1/8		
5994	G/A	V144I	3/8	DE loop	B-cell/MHC Class-I
6014	A/C	L150F	5/8	DE loop	B-cell/MHC Class-II
6038	C/T	—	1/8		
6039	A/G	—	1/8		
6046	G/A	—	1/8		
6051	C/A	—	1/8		
6172	A/G	—	1/8		
6206	G/A	—	1/8		
6222	A/G	—	1/8		
6405	G/A	—	4/8		
6417	A/G	—	4/8		
6435	T/C	—	4/8		
6440	A/C	—	1/8		
6441	A/C	—	1/8		
6459	G/A	—	1/8		
6459	G/T	—	1/8		
6460	G/A	—	1/8		
6497	T/G	—	1/8		
6497	C/T	—	1/8		
6540	A/G	I325M	6/8	*β*-G1	MHC Class-I/MHC Class-II
6642	G/A	—	4/8		
6689	C/A	T375N	2/8	HI loop	B-cell/MHC Class-I/MHC Class-II
6693	G/A	—	1/8		
6698	G/A	—	1/8		
6712	G/A	—	1/8		
6799	A/G	I412V	3/8	H2	MHC Class-I/VLP formation
6823	G/A	D420N	2/8	H3	B-cell/MHC Class-I/MHC Class-II/VLP formation
6828	C/A	N422D	4/8	H3	B-cell/MHC Class-I/MHC Class-II/VLP formation
6829	A/G	—	3/8		

(—) Synonymous variations with regard to the prototype reference D90400.1. T: threonine; N: asparagine; D: aspartic acid; V: valine; I: isoleucine; L: leucine; F: phenylalanine; M: methionine.

**Table 8:** HPV58 LCR sequence variation with regard to the reference sequence D90400.1. The table also shows the transcriptional factor binding sites in the nucleotide altered.

Genome position	Nucleotide variation	Number of isolates	Transcriptional factor
7150	G/T	1/6	
7169	Del TATACAT^*^	1/6	
7187	T/C	1/6	
7188	T/A	1/6	
7189	A/C	1/6	
7189	Del A	1/6	
7188	Del TATGT	1/6	
7197	G/C	1/6	
7202	C/T	1/6	
7210	T/A	3/6	
7233	G/A	1/6	
7244	C/A	1/6	YY1
7268	C/G	1/6	YY1
7269	C/T	2/6	YY1
7283	Ins TGTCAGTTTCCT	1/6	
7299	C/G	3/6	
7319	A/G	1/6	
7347	G/A	1/6	Oct-1, C/EBPbeta
7360	T/C	1/6	NF-1
7384	T/G	1/6	C/EBPbeta
7410	G/A	1/6	
7436	G/A	1/6	Oct-1, C/EBPbeta
7444	T/G	1/6	
7446	T/G	1/6	
7450	A/G	1/6	
7498	T/G	1/6	
7537	T/C	2/6	
7701	G/A	1/6	
7729	A/C	1/6	
7739	A/G	2/6	
7745	C/A	3/6	NF-1
7760	G/C	1/6	AP-1
7760	A/G	1/6	AP-1
7793	T/C	1/6	E2
7794	A/G	3/6	E2

Del: deletion,Ins: insertion. ^*^Novel HPV58 deletion.
